# Illinois State Dental Society

**Published:** 1888-08

**Authors:** C. N. Johnson


### ILLINOIS STATE DENTAL SOCIETY.

### TWENTY-FOURTH ANNUAL MEETING.

REPORTED FOR THE INDEPENDENT PRACTITIONER.

BY C. N. JOHNSON, L. D. S., D. D. S.

CONTINUED FROM PAGE 370.

### THURSDAY AFTERNOON SESSION.

The next in order was Dr. J. D. Moody's paper on "Some main points touching the conservative treatment of teeth whose pulps are nearly or quite exposed."

In this paper the essayist confined himself to those pulps which were nearly exposed and gave his method of treating them. The majority of teeth with which we have to do are of faulty organization. The characteristic white, chalky, friable enamel, disintegrated dentine, and a leathery mass occupying the bulk of the cavity, tell the story with which we are all so familiar. As a text, we will suppose a cavity on the distal surface of a cuspid, bicuspid or molar, in a tooth like the above. An impacted metal filling in such a case is incompatible with tooth structure, and is the worst material under these circumstances to employ at first. In some cases we might use an oxyphosphate, with tin and gold over it, but in many instances recurrence of decay will take place. The factors are: lowered vitality, frail tooth structure, micro-organisms, and a sensitive pulp.

He outlined his treatment of these cases as follows: "Where inflammation exists, first subdue it by any of the known remedies used for this purpose. Then use some kind of filling which is non-irritant, moisture tight, a non-conductor, compatible with tooth structure, and with some degree of permanency. These qualities are found only in the cements and gutta-percha. First apply the rubber-dam, to insure dryness. Then, in excavating leave a portion of dentine over the pulp-chamber, but be sure that you have clean margins. The essential point in these operations is to pack a line of gutta-percha along the cervical margin of the cavity, trimming it down even with the edges. Then complete the filling with oxyphosphate, and coat it all with chloro-percha or sandarac varnish till it crystallizes. By the use of gutta-percha you prevent disintegration of the oxyphosphate at the cervical margin. Leave this in from six months to two years; then you can usually refill with metal. The reasons for this treatment are, that in time teeth become harder, and will resist decay and bear contact with metal fillings better; and this hardening and the formation of secondary dentine renders the tooth more capable of bearing impact than before. Oxyphosphate is non-irritant to a pulp in near exposures, or so only in rare cases."

Mastic varnish has been advocated as a protection to the pulp before putting in oxyphosphate, but in the use of this material the pulp does not need protection, and the use of the varnish precludes the very thing for which the cement is used, viz., its therapeutical action upon both the pulp and tooth substance. Oxyphosphate is the best material for this purpose. It is tolerated by tooth structure, and by the pulp if not quite exposed, better than anything with which he was acquainted.

In treating on this subject the essayist was led to compare notes with Dr. W. D. Miller, who, in an article in the INDEPENDENT PRACTITIONER for February, 1885, condemns oxyphosphate as being unreliable for a temporary filling, stating that, as a rule, in a short time it will be dissolved out at the cervical margin, and that secondary caries advances with rapidity, because the antiseptic properties of oxyphosphate are almost zero. He also affirms that the smallest amount of moisture in the dentine prevents thorough adaptation of the cement. On page 62 he says:

"We need in a cement a material which may be used in very soft, porous teeth, sometimes over partially decalcified, moist dentine, and which will not only stop decomposition of softened dentine under the filling, but will also effectually shut out external agents, and at the same time, if possible, incite the underlying dentine to a recalcification. In all these points the oxyphosphates are wanting, besides giving very inconstant results in the most favorable cases."

Now, the essayist found that in almost every one of these cases cited his experience has been exactly the opposite, and believed that Dr. Miller's failures have been due to the fact that he did not use gutta-percha at the cervical margin.

He quoted 524 cases which have come under his observation in the last year, and all these of the extreme character spoken of in the beginning of the paper. Of these 243 were left one year and then filled permanently. In 230 of these the pulp was in good condition; five contained dead pulps where he did not expect them, and eight where they were marked doubtful. Those left about two years and then filled permanently numbered 190. Of these 179 were in good condition, six contained dead pulps where not expected, and five where marked doubtful. The remaining 91 cases, left over two years, the average time being three and a half years each, have in the majority been replaced with metal fillings, the remainder being in good condition yet.

He does not claim great permanency for oxyphospate fillings, but by the use of this material these frail teeth become harder, the pulp is better protected, and the tooth placed in a suitable condition for the insertion of a permanent filling. Altogether, it is given more years of serviceability than by any other means.

Session adjourned.

THURSDAY EVENING.

Dr. W. A. Johnston read a paper on "What Shall We Do with Inflamed Pulps?" The following is an abstract:

The question, "What shall we do with inflamed pulps?" is much like the one, "What shall we do with our daughters?"—it continually confronts us. The man who has no failures in treating inflamed pulps probably has no practice. Inflammation in a pulp is the same as inflammation in other tissues in its symptoms and sequelæ, and may terminate either by resolution or putrefaction; but it is unlike other inflammations on account of its position, having unyielding surroundings. It also lacks absorbents to carry away inflammatory products, and the blood supply may be cut off. The brain is contained in a long box, and inflammation of the brain is very dangerous, but even this has openings through which the membranes and cerebro-spinal fluid may protrude, and thus relieve the pressure. The tooth has no such safety-valve; an inflamed pulp means toothache.

In most cases destruction of the pulp is necessary, but if there is only slight trouble from pulp stones we may reduce it by counter irritation on the gums. If the pulp is acutely inflamed, open into the pulp chamber to give relief.

First remove the cause; then, when the pain subsides, give a saline cathartic for systemic treatment. In using iodine for counter irritation, do not use a steel instrument to apply it; wind a little cotton on a match-stick—and you will use iodine, not iodide of iron. If remedies fail to relieve the trouble, then devitalize. First quiet the pain with some one of the stimulating, warm tasting, volatile drugs, before applying arsenic. If this medicine will also remove the exudate, it is better. A continuous current of warm air on a nearly exposed pulp will relieve sensibility, and if our remedies are warmed, we will save suffering.

If we can persuade the blood to leave the crowded territory, all will be well, but otherwise the white blood corpuscles will work out of the vessels and die, and suppuration begins. For diverting the blood, stimulants are indicated.

If destruction of the pulp is necessary, we should not previously apply escharotics, on account of forming an eschar through which the arsenic cannot act with certainty.

It is good to administer quinine in the cases just described; it has a tonic effect on the nervous system. Especially is this necessary in malarial districts, and then a full dose should be given. I would not introduce a permanent filling over a pulp which had been recently inflamed; if trouble occurs, it is more difficult of removal.

The best method of destroying pulps is to relieve the pain, remove the bloodclots and coagula, and apply arsenic one part, hydrochlorate of cocaine four parts, and lanolin five parts, allowing the application to remain 24 hours.

Inflammation of the pulp seldom yields to cocaine, but when it is combined with lanolin the effect is sometimes magical. The essayist applied this once to an aching molar in a boy of fifteen, sealing it with gutta-percha. The pain subsided, and the following day he removed the pulp painlessly. In these cases the pulp comes out as though greased.

Pain on occlusion in an inflamed tooth can be relieved by holding a wooden toothpick between the teeth of the opposite side.

Capping a pulp which has once been inflamed is doubtful practice. Patients lose confidence in us if we fail, even though we may have done our best. With our facilities in the way of instruments for removing pulps, it is better to destroy. In any of these operations we must be careful and thorough.

Patients come to us for three reasons—prudence, pride, or pain. The latter is most often the incentive, and we must study to relieve it. The cases where pulp capping would be the most successful are those in which the patients are young, black-haired, and healthy.

The papers of Dr. Moody and Dr. Johnston were discussed together.

DISCUSSION.

*Dr. A. W. Harlan*—In opening this discussion, I would say in the first place that the essayist has treated the subject in a manner fitting its importance. In answer to the question, "What shall we do with inflamed pulps?" I cannot agree that all should be destroyed. Pulps in young persons' teeth should be saved if possible. It would be bad practice to destroy pulps in the first permanent molars at seven or eight years; also in the central incisors. They should not be destroyed before the teeth have attained their growth. Pulps of teeth that are inflamed should not always be destroyed in adult life. In the anterior part of the mouth it sometimes causes disfigurement on account of discoloration, especially in women.

I have little hope of the permanency of capping in adults, where the pulp has been inflamed for any length of time, and where, when the pulp is probed, pus follows, though in vigorous patients the pulp may live.

The necessity for retaining pulps is not so great after the twenty-fifth year, when the teeth have attained their development. The teaching of the paper is good, and the combination of arsenic, cocaine and lanolin is efficacious in destroying pulps. The essayist did not mention the value of a blister as a counter irritant; it should not be lost sight of. I concur in the statement that remedies should be warmed before applying to a pulp. In cases where it is undesirable to destroy an inflamed pulp, use agents to reduce the local inflammation and reduce the blood pressure, such as counter irritants. In general, systemic treatment is of little use, owing to the bony canals surrounding the pulp. It is a good practice to puncture the pulp carefully to bring relief. Apply iodoform or iodol, and seal the cavity without pressure. After the pulp has been punctured and sealed for a week, I do not favor further application of remedies that will destroy the surface of the pulp. The least irritation the better. I would not advocate any immediate capping, but would dress temporarily and allow to remain a few weeks.. Many failures result from too much haste, and from the manner of introducing the material. It should be inserted so as not to cause pressure.

Inflamed pulps in young persons may be saved by the plan of treatment given by the essayist, or that which I have proposed.

*Dr. Crouse*—I was in hopes Dr. Harlan would review the other paper. I am somewhat disappointed with the views of Dr. Moody, and also with his treatment. I shall be obliged to take the opposite side. I have no faith in this theory of incompatibility; I never could understand it. I do know that dentine under tin and gold will sometimes grow hard. One provision of nature is to restore destruction of parts, and under favorable circumstances it will do it. I have in my own mouth two cavities in which no filling has been inserted. The decay progressed to a certain point and then stopped, and it is now hard, and the dentine will shine when cut. I do not object to the use of temporary fillings, but I take issue with Dr. Moody in his selection of materials. If tin is ever preferable to use in the mouth, it is in these cases. It is better than any of the oxyphosphates or oxychlorides. Amalgam is better than either. Oxyphosphates and oxychlorides are too temporary. The essayist recommended lining the cervical margin with gutta-percha. That is good, but it is better to use it in the whole cavity.

The best application for an exposed or nearly exposed pulp is carbolic acid, saturated solution. It forms a coagulum. Then apply oxychloride of zinc, and you have the best capping possible. This must be done carefully; it is the most delicate operation in dentistry. I have employed this method for eighteen years; I have frequently tried others, but always go back to the old plan. I would strongly advocate a careful trial of my method. Do not try it simply to prove its fallacy, but go about it conscientiously, to honestly find out if there is anything in it. I have had less failures with it than with any other. Some of the pulps may die, but ordinarily you never hear from them, as the teeth do not discolor and there is no disturbance. Sometimes the patient will tell you there has been a twinge of pain, but that is all. The pulps which die under this treatment become mummified, and cause no trouble. You should always explain to patients the chances taken in capping.

As to treatment in the way of filling, as I have said, I should use gutta-percha for the entire filling, rather than any oxyphosphate or oxychloride. I was pleased with the manner in which Dr. Moody stated and kept a record of his cases; I have kept one eighteen years. In capping, the mistake is sometimes made of using too small a cap. Let it extend well around the exposure.

*Dr. Wassall*—Years ago all dentists capped pulps with oxychloride, with more or less success. Later on, oxyphosphate was introduced, and operators had greater success. Dr. Crouse has been peculiarly successful with oxychloride, but I think he could be more successful with oxyphosphate. There is one phase of pulp disease which is very difficult of diagnosis. It is the first stage of inflammation, known as hyperæmia, or simple congestion. The pain is usually obscure and difficult to locate. It may cause neuralgic pains on one side of the head, and if so, the offending member will be found on the same side. A case recently came under my notice where the patient complained of pain on one side, and in searching for the seat of trouble I found several large fillings, all in good condition, with living pulps. Apparently there was nothing to point out which tooth caused the disturbance; but on tapping them I found a slight soreness in one, and this gave an index to the trouble. The heat test is not always reliable in proving the condition of the pulps, as you will often get a response when the pulp is dead, on account of the gas expanding in the pulp-chamber, and causing pressure at the apex. Then again, a live pulp will not always respond to heat, so that percussion is the best test.

*Dr. Gilmer*—I have used something for counter-irritation where pulps are inflamed, that takes the place of capsicum plasters. I procure a common mustard plaster, cut it up into pieces of a suitable size and use them.

In capping pulps in anterior teeth, room is a consideration. You cannot use a large capping. I would place a small piece of quill in position and flow oxyphosphate over it. The quill will not be destroyed.

Dr. Wassall spoke about the unreliability of heat in testing a pulp. If he had used cold, he would have succeeded better.

*Dr. Cushing*—What has been said has mostly covered the ground. The papers were good. I must emphasize Dr. Crouse's advocacy of oxychloride in capping pulps. We capped many pulps in the beginning that should not have been attempted, and at first they seemed to do well, but there came a time when they did not seem so successful. Then we believed that oxychloride was too much of an irritant, and accepted oxyphosphate as the proper material. We used it till we became convinced that it was not so good as oxychloride. More pulps die from the effects of oxyphosphate than from oxychloride, so that I have abandoned it and use the latter.

Dr. Crouse stated that capping a pulp is a most delicate operation. It is so. Many failures result from causing pressure on the pulp, but with oxychloride it is possible to cap a pulp to any thickness without pressure, by flowing the material over it in a very liquid form. It requires longer to set, but in these cases it is better to take time. Ordinarily, young men are too hasty in filling teeth permanently.

It may seem strange to the younger members to hear so great a diversity of opinion, but we differ in manipulation and judgment, and you must not be confounded when you hear different reports.

It is sometimes absolutely necessary to endeavor to save a pulp. I apply copal ether varnish previous to flowing the oxychloride over the pulp, and in nine cases out of ten there is no pain.

*Dr. Morrison*—I want to add my testimony to Drs. Crouse and Cushing. In 1861 I had an exposed pulp, and the best dentists advocated the removal of the tooth on account of the crowded condition of the teeth and the large exposure of the pulp. They said it could not be saved alive. That pulp was capped with oxychloride, which caused most intense pain for a moment. It was then filled with gold, which remained in fifteen years, and I am now wearing a platinum shell over the tooth. It is the most sensitive tooth in my mouth to-day.

My method in capping is to use wood creosote in the cavity for a minute; then absorb the excess, and apply the oxychloride thin. I use a French bibulous paper to follow up the material, and it absorbs the moisture and dries the oxychloride.

Sometimes an inflamed pulp, under a filling, will cause deep-seated aches on the side of the jaw, especially at this season of the year, and in malarial districts. The patients cannot point out the tooth, and it is hard for the dentist to do it till about ten days after the trouble begins. Then ordinarily some one tooth will respond to tapping. Usually these troubles will pass off if the patient has patience.

*Dr. Harlan*—There is scarcely any more important subject in dentistry. We should take proper preliminary precaution in treating these cases. Use the rubber-dam; and another point is to cleanse the mouth with properly tempered water. Again, after it has been determined advisable to cap, you should study to have the filling covering the cap made of such a shape that you can enter the pulp-canal without disturbing the filling in case trouble follows.

The best thing Dr. Cushing said was when he recommended copal ether varnish. It is non-irritant, unyielding, and prevents the probability of pain from the oxychloride. If the pulp is healthy, it is not so important what we use. In these operations we must be extremely careful; but if we are honest and conscientious, we may save many pulps that are destroyed.

*Dr. Sitherwood*—Instead of using the mustard plaster recommended by Dr Gilmer, I buy the regular capsicum plaster, and cut it up into suitable sizes. It is thinner than the pads we get for this purpose.

*Dr. Keith*—I would like to ask what is the advantage of using oxychloride over oxyphosphate when the pulp is capped with copal varnish ?

*Dr. Noyes*—It always seemed to me difficult to understand why an inflamed or exposed pulp should be treated differently from any other wound. The essentials of success in the treatment of exposed pulps are : First, cure the trouble; second, render the cavity and contents aseptic ; third, protect the pulp perfectly without irritation, and shield it from heat or cold. If we want success in these cases, we must recognize the fact that septic poisoning is always possible in the mouth. These essentials can be secured by a variety of methods, where the operator is skillful and the opportunity favorable. The treatment may be thus summed up : Removal of irritants, protection of the pulp from further irritation and counter-irritation.

You can gain antisepsis better by non-escharotic than by escharotic means. There is no reason for irritating or producing an eschar on the surface of the pulp after the disease had been cured. It may be necessary, to induce a cure, but usually it will yield to milder methods.

*Dr. A. W. Freeman*—I have never seen a tooth with the pulp alive that would not respond to hot gutta-percha.

*Dr. Townsend*—I have seen many cases where I could not get a response from gutta-percha, but have always been successful with rhigolene spray. I have capped pulps with oxyphosphate and they are living.

*Dr. Taggart*—Some gentlemen condemn the use of carbolic acid in certain places because it will coagulate albumen, while they advocate bichloride of mercury for the same cases. Now, the latter will also coagulate albumen, if chemistry is right.

*Dr. Cummins*—My method of treating exposed pulps is as follows: After excavating thoroughly I apply creosote for half a minute. I then use a formula of cocaine, chloroform, morphia, and oil of cajeput. After this is applied I use spunk with one side coated with copal ether varnish, and let it remain six or eight hours. I then cap with the varnish over the spunk, and if at that time the pulp is all right, I fill with gold or amalgam. If not, I puncture the pulp and use olive oil and glycerine, one to six.

*Dr. Stevens*—I am glad so many are succeeding; I have never saved an exposed pulp by these methods.

Subject passed.

(TO BE CONTINUED.)

